# Mind the gaps: age and cause specific mortality and life expectancy in the older population of South Korea and Japan

**DOI:** 10.1186/s12889-020-08978-x

**Published:** 2020-06-01

**Authors:** Myunggu Jung, Woorim Ko, William Muhwava, Yeohee Choi, Hanna Kim, Young Su Park, Gizachew Balew Jambere, Youngtae Cho

**Affiliations:** 1 Faculty of Epidemiology and Population Health, London School of Hygiene & Tropical Medicine, London, UK; 2 Department of Public Health Science, Graduate School of Public Health, Seoul National University, Seoul, South Korea; 3 African Centre for Statistics, United Nations Economic Commission for Africa, Addis Ababa, Ethiopia; 4 Department of Social Welfare, Graduate School of Social Welfare, Ewha Womans University, Seoul, South Korea; 5 Department of Anthropology, University College London, London, UK; 6 EngenderHealth-Ethiopia, Addis Ababa, Ethiopia

**Keywords:** Population ageing, Decomposition, Life table, Longevity, Old-age life expectancy

## Abstract

**Background:**

Recent life expectancy gains in high-income Asia-pacific countries have been largely the result of postponement of death from non-communicable diseases in old age, causing rapid demographic ageing. This study compared and quantified age- and cause-specific contributions to changes in old-age life expectancy in two high-income Asia-pacific countries with ageing populations, South Korea and Japan.

**Methods:**

This study used Pollard’s actuarial method of decomposing life expectancy to compare age- and cause-specific contributions to changes in old-age life expectancy between South Korea and Japan during 1997 and 2017.

**Results:**

South Korea experienced rapid population ageing, and the gaps in life expectancy at 60 years old between South Korea and Japan were reduced by 2.47 years during 1997 and 2017. Decomposition analysis showed that mortality reductions from non-communicable diseases in South Korea were the leading causes of death contributing to the decreased gaps in old-age life expectancy between the two countries. More specifically, mortality reductions from cardiovascular diseases (stroke, ischaemic and hypertensive heart disease) and cancers (stomach, liver, lung, pancreatic cancers) in South Korea contributed to the decreased gap by 1.34 and 0.41 years, respectively. However, increased mortality from Alzheimer and dementia, lower respiratory tract disease, self-harm and falls in South Korea widened the gaps by 0.41 years.

**Conclusions:**

Age- and cause- specific contributions to changes in old-age life expectancy can differ between high-income Asia-pacific countries. Although the gaps in old-age life expectancy between high-income Asia-pacific countries are primarily attributed to mortality changes in non-communicable diseases, these countries should also identify potential emerging threats of communicable diseases and injuries along with demographic ageing in pursuit of healthy life years in old age.

## Background

Recent life expectancy gains in high-income Asia-pacific countries, as classified by the IMF, including Australia, New Zealand, Japan, Hong Kong SAR, Republic of Korea, Taiwan Province of China and Singapore, are largely the result of enhanced longevity at older ages, and this trend leads to rapid population ageing in those countries [1, 2]. Although the factors which explain life expectancy gains are multifaceted, previous studies have demonstrated that this has largely resulted from improvements in effectiveness and coverage of healthcare [3]. From a theoretical perspective, the ‘epidemiological transition theory’ relates changing patterns of population distributions to leading causes of death from one predominant group of infectious diseases in developing nations to what Abdel Omran referred to ‘*degenerative and man-made diseases*’ in developed nations [4].

Recent comparative studies of life expectancy and causes of death between high-income Asia-pacific countries showed that causes of death contributing to the changes in life expectancy can differ between them [5–7]. These studies, however, have tended to focus on changes in life expectancy within countries, while gaps in life expectancy between countries have received less attention. Exploring the gaps in life expectancy between countries can enhance understanding of causes of the gaps in life expectancy and facilitate cross-national policy learning. A combined analysis of changes in life-expectancy within and gaps in life expectancy between high-income Asia-pacific countries is the first novelty of this study. The second novelty is that this study uses an elderly population-focused approach for a comparative study of cause of death and life expectancy. Previous studies have substantially concentrated on life expectancy at birth, but this approach may not necessarily correspond to life expectancy at an older age, or may overlook different mortality trajectories among the elderly population in high-income Asia-pacific countries. Therefore, this study provides new insights into the gaps in old-age life expectancy between high-income Asia-pacific countries in an era of global population ageing.

Among high-income Asia-pacific countries, the Republic of Korea (hereafter South Korea) and Japan have both experienced demographic ageing due to rapidly increasing longevity and declining fertility rates. As a result, Japan has been widely regarded as the most aged country in the world and South Korea, with the fastest ageing population in the world, is projected to take the lead in life expectancy from Japan between 2030 and 2040 [8]. Although South Korea and Japan share many features in terms of demographic changes, health policy directions and geography [8–15], there has been limited evidence from comparative studies of age- and cause-specific contribution to life expectancy among the elderly in the two countries. It is therefore of interest to closely compare age- and cause-specific contributions to the recent changes in life expectancy among the elderly between South Korea, the potential life expectancy leader, and Japan, currently first in life expectancy.

Age 65 is usually used as an old age threshold, because in some countries it is the age at which people could be eligible for a full state pension. However, in South Korea, normal pension age is now in the process of change. As of 2020, individuals over 62 years old were eligible for the normal pension in South Korea, and the criterion was 60 years old until 2012. In Japan, the old age basic pension benefits are currently paid from 65 years old, whereas the pension age was 60 years old in the 1990s. Since this study compares old-age life expectancy between the two countries in 1997 and 2017, this study defines older adults as aged 60 years or older. The aim of this study is therefore to report on a comprehensive comparative study of age- and cause-specific contributions to changes in life expectancy at 60 years between South Korea and Japan. This study focuses on population data between 1997 and 2017 and observes (1) age structural changes; (2) age-standardised mortality rates; and explores (3) age- and cause-specific contributions to increasing life expectancy at 60 years in South Korea and Japan and (4) age- and cause-specific contributions to decreasing gaps in life expectancy at 60 years between South Korea and Japan.

## Methods

### Data

**Fig. 1:**
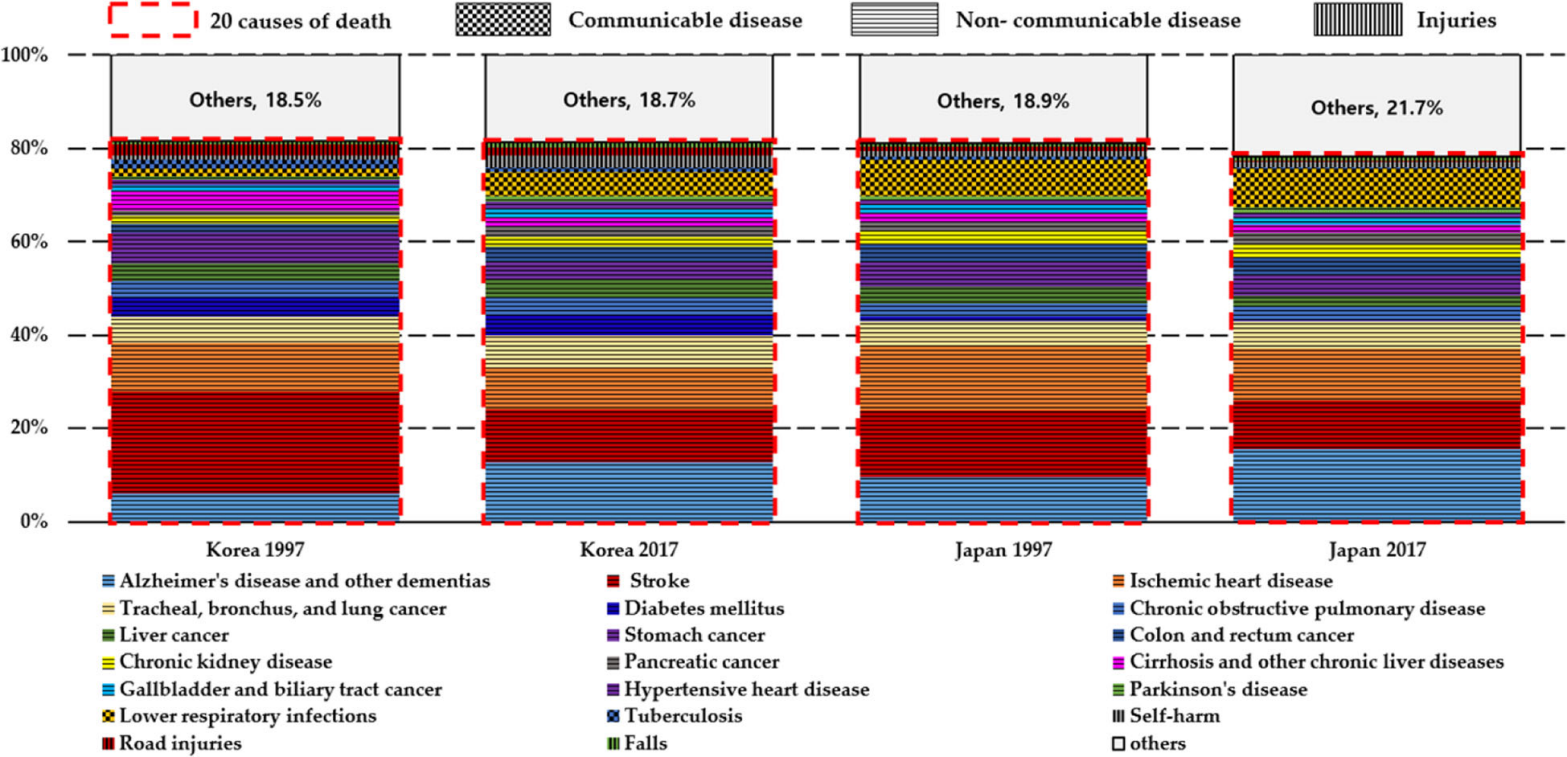
Proportion of top twenty causes of death for persons aged 60 years and older at IHME’s Level 3 category in South Korea and Japan between 1997 and 2017

This study obtained data of the South Korean and Japanese population by five-year age groups from the World Population Prospects (WPP) 2019, and age- and cause-specific deaths from the Global Burden of Disease (GBD) 2017 Study [16]. The World Population Prospects and GBD data are publicly available on websites of the World Population Prospects (https://population.un.org/wpp/) and the Global Health Data Exchange (http://ghdx.healthdata.org). The GBD 2017 study follows the Guidelines for Accurate and Transparent Health Estimates Reporting (GATHER), which includes recommendations on documentation of data sources, estimation methods, and statistical analysis. Detailed methods for the GBD 2017 study are provided in other publications [17]. The GBD 2017 study organises causes of death in a hierarchical list containing four levels. At the highest level (Level 1), all disease burdens are divided as three mutually exclusive and collectively exhaustive categories: communicable, maternal, neonatal, and nutritional (CMNN) diseases; non-communicable diseases (NCDs); and injuries. Level 2 distinguishes the Level 1 category into 21 cause groups, and Level 3 disaggregates these causes further, into 169 cause groups. The GBD data is available in the form of the number of deaths in point estimates and 95% uncertainty intervals. This study particularly used the mean values of the number of specific causes of death at Level 3 for both sexes in South Korea and Japan between 1997 and 2017. Among 169 causes of death, the top 20 causes of death for persons ages 60 years and older based on South Korea in 2017 were filtered out, and the remaining causes were merged into the *‘Others’* category. Figure 1 shows the top 20 causes of death in South Korea and Japan in 1997 and 2017. Although the ‘*Others*’ category slightly increased from 1997 to 2017 in both countries, the top 20 causes accounted for almost 80% of the total number of deaths in both countries for the two periods. In addition, the top 20 causes based on South Korea in 2017 were selected to explore the causes of death among the elderly in contemporary developed societies and investigate how South Korea caught up with the old-age life expectancy of Japan between 1997 and 2017.

### Analysis

A descriptive analysis of age structural changes in South Korea and Japan between 1997 to 2040 was carried out with the WPP 2019 data. Then, the multiple decrement table by five-year age groups for both countries between 1997 and 2017 was constructed to compute life expectancy (LE). A broad mathematical assumption of the multiple decrement life table is that an individual surviving to a certain age is the product of all independent risk-of-death probabilities. Although sampling variation is not an essential issue when calculating LE at national levels, we used Monte Carlo simulations using the probability of dying from an abridged life table to generate a binomial distribution of death numbers. The simulation was performed 10,000 times to generate the LE distribution, where the mean value was used as LE and the 2.5th and 97.5th percentiles of the distribution were used as the 95% confidence interval of LE. Calculation techniques for multiple decrement life tables and the standard error of LE are described elsewhere [18, 19].

In order to compare cause-specific mortality rates, this study uses age-standardised mortality rates per 1000 persons, with a direct method of standardisation further calculated to eliminate the effect of different age structures among the different population structures across two countries and times. The directly standardised mortality rate is given by the following formula;

$$ \mathrm{Directely}\ \mathrm{standardised}\ \mathrm{mortality}\ \mathrm{rate}=\frac{d_{it}}{p_i}\ast \left(\frac{p_{ir}}{N_r}\ast 1000\right) $$

where *d*_*it*_ is the number of deaths for each specific cause of mortality *t* at age *i*; *p*_*i*_ denotes the number of persons at age *i* in the observed population; *N*_*r*_ is the total number of persons in the reference population; *p*_*ir*_ denotes the number of persons at age *i* in the reference population. The population to be used as a reference was derived from the average of the age distributions of South Korean and Japan in 1997 and 2017 at each age group. In addition, the Chi-square test was calculated for testing the significance of differences in age-standardised mortality rates (ASMR) of the two countries between 1997 and 2017.

Pollard’s actuarial method of decomposing life expectancy was then used to estimate the age- and cause-specific contributions to changes in life expectancy [20, 21]. This method was selected because it allows for simultaneously decomposing both different age groups and different causes of death. The decomposition method was calculated to examine the difference in life expectancy of (1) South Korea between 1997 and 2017 and (2) Japan between 1997 and 2017, which is given by the formula;

$$ {e}_{60}^{2017}-{e}_{60}^{1997}={\sum}_{i=1}^n{\sum}_{x=60}^{\omega}\left(i{Q}_x^{1997}-i{Q}_x^{2017}\right)\ast {\omega}_x; with{\omega}_x=\frac{1}{2}\left(x{P}_{60}^{2017}{e}_x^{1997}+x{P}_{60}^{1997}{e}_x^{2017}\right). $$

where ${e}_x^{2017}$ and ${e}_x^{1997}$ are the life expectancies at age *x* in 2017 and 1997 for the the decomposition of life expectancy within countries; *n* denotes the number of causes of death; $i{Q}_x^{2017}$ and $i{Q}_x^{1997}$ is the mortality rate of the *i* cause of death at age interval *x* with the weight ω_x_. The values $x{P}_{60}^{2017}$ and $x{P}_{60}^{1997}$ denote the probability of surviving from 60 years old to 60 + *x* years of age between different times.

The same decomposition method was further calculated to examine the difference in life expectancy between (3) South Korea and Japan in 1997, and (4) South Korea and Japan in 2017, which is given by the following formula;

$$ {e}_{60}^j-{e}_{60}^k={\sum}_{i=1}^n{\sum}_{x=60}^{\omega}\left(i{Q}_x^k-i{Q}_x^j\right)\ast {\omega}_x; with{\omega}_x=\frac{1}{2}\left(x{P}_{60}^j{e}_x^k+x{P}_{60}^k{e}_x^j\right). $$

where *j* corresponding to Japan and *k* corresponding to South Korea for the decomposition of life expectancy between countries with the weight ω_x_ between different countries. This study based all life expectancy estimates on the population aged 60-64. 

## Results

### Rapid population ageing and fertility declined in South Korea

**Fig. 2:**
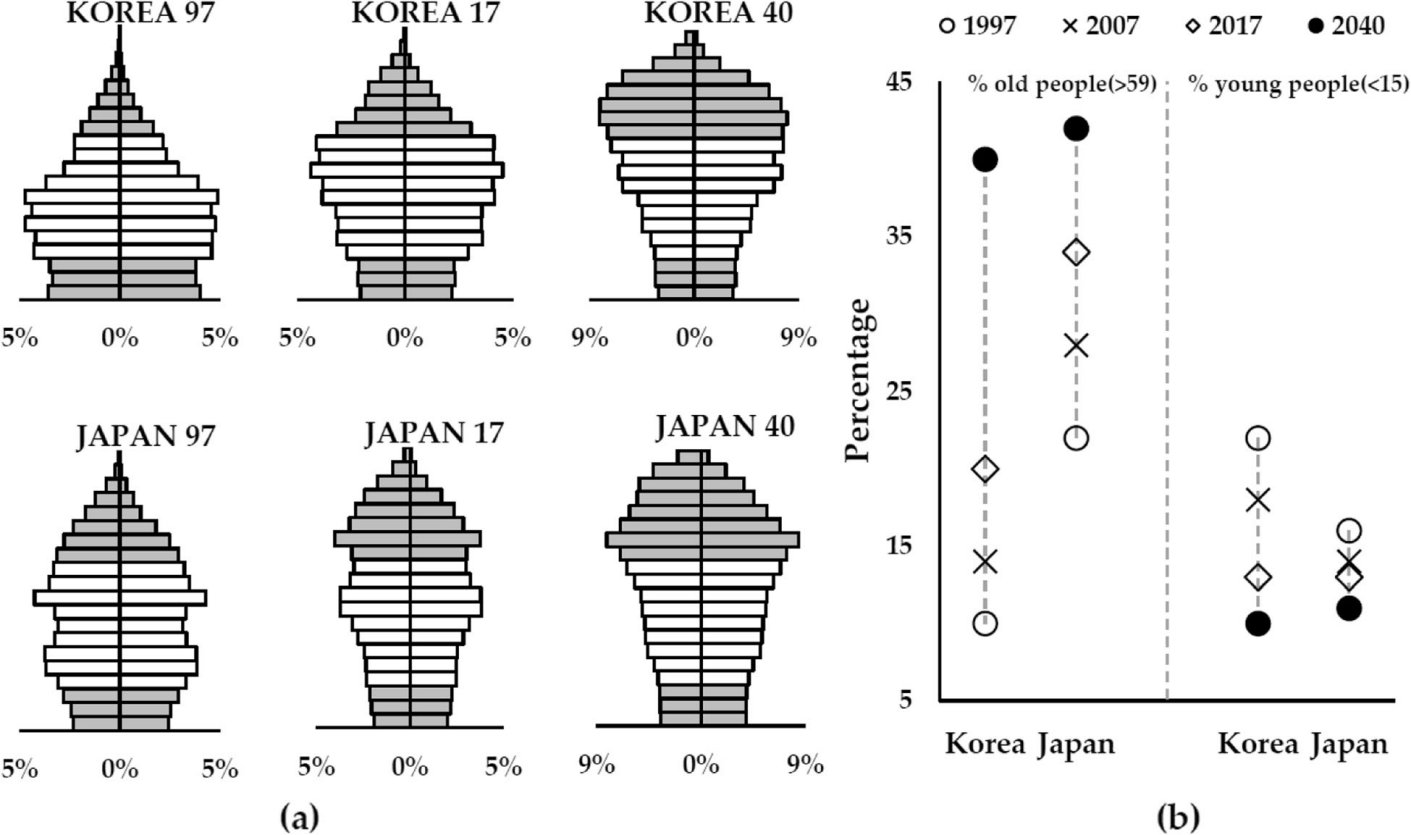
Population pyramids **a** and proportions of old and young age groups **b** of South Koreans and Japanese between 1997 and 2040

Figure 2 depicts the UN medium variant projection estimates of age structural changes in South Korean and Japan [1]. The proportion of elderly population over 60 years of age increased in both countries from 1997 to 2017 and is projected to further grow in 2040, as shown in the population pyramids in Fig. 2a (top grey bars). Although Japan had larger proportions of the population over 60 years of age in 1997 and 2017, the proportion in 2040 is expected to be more or less equal between the two countries, 40% in South Korea and 42% in Japan. In terms of the pace of population ageing, while Japan seems to follow a gradually increasing trend in proportions of the population over 60 years old, South Korea seems to follow a steadily increasing trend between 1997 and 2040 (see Fig. 2b). Particularly, the projected increase from 2017 to 2040 in the proportions of the population over 60 years old in South Korea is striking, as South Korea is expected to catch up with Japan’s four decades of growth just in the next two decades. Furthermore, the proportions of the population under 15 years old (bottom grey bars in Fig. 2 a) in South Korea shrank between 1997 and 2017 at a much faster pace than in Japan. Hence, in comparison with Japan, South Korea experienced faster population ageing, accompanied by precipitous falls in birth rates; and hence the country is very likely to face much more pressing demographic challenges in the next 20 years.

### Changes in life expectancy and age-standardised mortality rates in older people

Although both countries experienced increases in old-age life expectancy between 1997 and 2017, South Korea rapidly caught up with life expectancy in Japan(see Table 1). Life expectancy at age 60 was 20.09(±0.04) years for South Korea and 23.87(±0.02) years for Japan in 1997, whereas the gap narrowed in 2017, as life expectancy increased to 25.09(±0.03) years for South Korea and 26.40(±0.01) years for Japan. The gaps in old-age life expectancy between the two countries therefore decreased by 2.47 from 3.78 in 1997 to 1.31 in 2017.

**Table 1:** Life expectancy at 60 years and older of South Korea and Japan in 1997 and 2017

Age group	1997 (95% CI)		2017 (95% CI)		Gaps in LE between countries		Changes in gaps between 2017 and 1997
	Korea (a)	Japan (b)	Korea (c)	Japan (d)	1997 (b-a)	2017 (d-c)	
**LE 60**	20.09 ± 0.04	23.87 ± 0.02	25.09 ± 0.03	26.40 ± 0.01	3.78	1.31	−2.47
**LE 65**	16.29 ± 0.04	19.77 ± 0.02	20.74 ± 0.03	22.15 ± 0.01	3.48	1.41	−2.07
**LE 70**	12.74 ± 0.04	15.93 ± 0.02	16.60 ± 0.03	18.04 ± 0.02	3.19	1.44	−1.75
**LE 75**	9.66 ± 0.04	12.35 ± 0.02	12.82 ± 0.02	14.18 ± 0.01	2.69	1.36	−1.33
**LE 80**	7.11 ± 0.04	9.19 ± 0.02	9.48 ± 0.03	10.67 ± 0.01	2.08	1.19	−0.89
**LE 85**	5.10 ± 0.04	6.61 ± 0.02	6.75 ± 0.03	7.69 ± 0.02	1.51	0.94	−0.57
**LE 90**	3.57 ± 0.06	4.63 ± 0.02	4.71 ± 0.04	5.30 ± 0.02	1.06	0.59	−0.47
**LE 95**	2.61 ± 1.00	3.14 ± 0.04	3.31 ± 0.06	3.45 ± 0.02	0.53	0.14	−0.67

Table 2 shows estimated age-standardised mortality rates (ASMRs) per 1000 persons for the population aged 60 years and older in South Korea and Japan between 1997 and 2017. Between 1997 and 2017, age-standardised all-cause mortality rates decreased from 47.9 to 27.4 in South Korea and from 31.2 to 23.4 in Japan. The comparative mortality figure (CMF) in Table 2 is defined as the expected number of deaths in the standard population compared with those observed [22]. The CMF shows that in 1997 all-cause mortality of the elderly population in South Korea was 54% higher than in Japan, but the figure decreased to 17% in 2017. The last column shows the changes in ASMRs of each cause from 1997 to 2017, where negative figures indicate falls and positive signs indicate rises in ASMRs over the periods. ASMRs from stroke and ischemic heart disease largely declined in both countries. The highest ASMRs at old ages in 1997 were observed in stroke in both countries, but they were Alzheimer’s disease and dementia for both countries in 2017. On the other hand, ASMRs rose from a few causes between 1997 and 2017. While only one cause in Japan 2017, pancreatic cancer, had higher ASMRs than those in 1997, South Korea experienced increased ASMRs from four causes: Parkinson’s disease, lower respiratory tract disease, self-harm and falls. Interestingly, the increased figure in Japan was only categorised in non-communicable disease within the IHME’s Level 1 category; however, the increased figures of the four causes in South Korea were categorised within all the three Level 1 classifications, Communicable, Non-Communicable disease and Injuries. Moreover, ASMRs of lower respiratory tract disease in Japan were higher than South Korea in both years, but the two countries had opposite trends, from 2.6 to 1.9 in Japan, from 0.9 to 1.5 in South Korea. The Chi-square test showed that difference in ASMRs between 1997 and 2017 was statistically significant in South Korea, which was primarily derived from mortality declines in non-communicable disease.

**Table 2:** Age- standardised mortality rates for 20 causes of death among older adults in South Korea and Japan between 1997 and 2017

Cause of Death	ASMR per 1000 (95% CI)				Differences between 1997 and 2017	
	1997		2017		Korea	Japan
	Korea	Japan	Korea	Japan		
**Non-communicable disease**	**35.2 (32.3–38.4)**	**21.8 (21.4–22.4)**	**19.2 (16.8–22.2)**	**15.6 (14.8–16.5)**	−15.9*	**−6.2**
Stroke	10.4(9.7–11.0)	4.6(4.5–4.6)	3.1(2.8–3.5)	2.4(2.3–2.5)	−7.2	−2.2
Ischaemic heart disease	5.5(5.0–6.0)	4.3(4.3–4.4)	2.6(2.3–3.0)	2.6(2.5–2.7)	− 2.8	−1.8
Stomach cancer	2.6(2.4–2.8)	1.6(1.6–1.6)	0.9(0.8–1.1)	1.0(1.0–1.1)	−1.7	−0.6
Cirrhosis / liver diseases	1.6(1.4–1.8)	0.5(0.5–0.5)	0.5(0.4–0.6)	0.4(0.4–0.5)	−1.1	−0.1
Chronic obstructive pulmonary disease	1.8(1.6–2.1)	0.9(0.8–0.9)	1.1(1.1–1.3)	0.6(0.6–0.7)	−0.7	−0.3
Liver cancer	1.5(1.3–1.6)	1.0(1.0–1.0)	0.9(0.8–1.1)	0.6(0.5–0.7)	− 0.6	− 0.4
Tracheal / lung cancer	2.2(2.0–2.4)	1.6(1.6–1.7)	1.7(1.5–1.9)	1.5(1.4–1.5)	− 0.5	− 0.1
Diabetes mellitus	1.6(1.5–1.8)	0.2(0.2–0.2)	1.1(0.9–1.3)	0.1(0.1–0.1)	− 0.5	− 0.1
Alzheimer / dementia	4.3(4.1–4.5)	3.4(3.3–3.4)	4.0(3.7–4.4)	3.3(3.1–3.4)	− 0.3	− 0.1
Gallbladder cancer	0.6(0.5–0.7)	0.5(0.5–0.5)	0.4(0.4–0.5)	0.4(0.4–0.4)	− 0.2	− 0.1
Hypertensive-heart disease	0.8(0.6–1.1)	0.3(0.3–0.5)	0.5(0.4–0.8)	0.2(0.2–0.4)	− 0.2	− 0.1
Chronic kidney disease	0.7(0.6–0.8)	0.9(0.9–0.9)	0.6(0.5–0.7)	0.6(0.6–0.7)	− 0.1	− 0.3
Pancreatic cancer	0.5(0.5–0.6)	0.6(0.6–0.6)	0.5(0.4–0.8)	0.6(0.6–0.7)	− 0.1	0.1
Colon and rectal cancer	0.8(0.8–0.9)	1.1(1.1–1.1)	0.8(0.7–1.0)	1.0(1.0–1.1)	0	−0.1
Parkinson’s disease	0.3(0.3–0.4)	0.3(0.2–0.3)	0.3(0.2–0.4)	0.3(0.2–0.3)	0.1	0.0
**Communicable disease**	**1.9(1.6–2.1)**	**2.8(2.7–2.9)**	**1.7(1.5–2.1)**	**1.9(1.8–2.0)**	**− 0.1**	**− 0.9**
Tuberculosis	0.9(0.8–1.1)	0.2(0.2–0.2)	0.3(0.2–0.3)	0.1(0.1–0.1)	− 0.7	− 0.1
Lower respiratory tract	0.9(0.8–1.0)	2.6(2.5–2.7)	1.5(1.3–1.7)	1.9(1.8–2.0)	0.6	− 0.7
**Injuries**	**1.5(1.4–1.7)**	**0.8(0.8–0.8)**	**1.3(1.1–1.6)**	**0.6(0.6–0.6)**	**−0.1**	**− 0.2**
Road injuries	0.8(0.8–0.9)	0.2(0.2–0.2)	0.4(0.3–0.4)	0.1(0.1–0.1)	− 0.4	− 0.1
Falls	0.3(0.3–0.4)	0.2(0.2–0.2)	0.3(0.3–0.4)	0.2(0.2–0.2)	0.1	−0.1
Self-harm	0.4(0.3–0.4)	0.4(0.4–0.4)	0.6(0.5–.0.7)	0.3(0.3–0.3)	0.3	−0.1
**others**	**9.3(7.5–11.3)**	**5.7(5.2–6.3)**	**5.0(3.8–6.2)**	**5.2(4.5–5.9)**	**−4.3**	**− 0.5**
**Total**	**47.9 (42.9–53.5)**	**31.2 (30.0–32.4)**	**27.4 (23.3–32.0)**	**23.4. (21.7–25.1)**	−20.4*	**−7.8**
**CMF**	**1.54**	**1.00**	**1.17**	**1.00**		

Note: * significant at 10% level

### Age- and cause-specific contributions to increasing old age life expectancy within countries

**Fig. 3:**
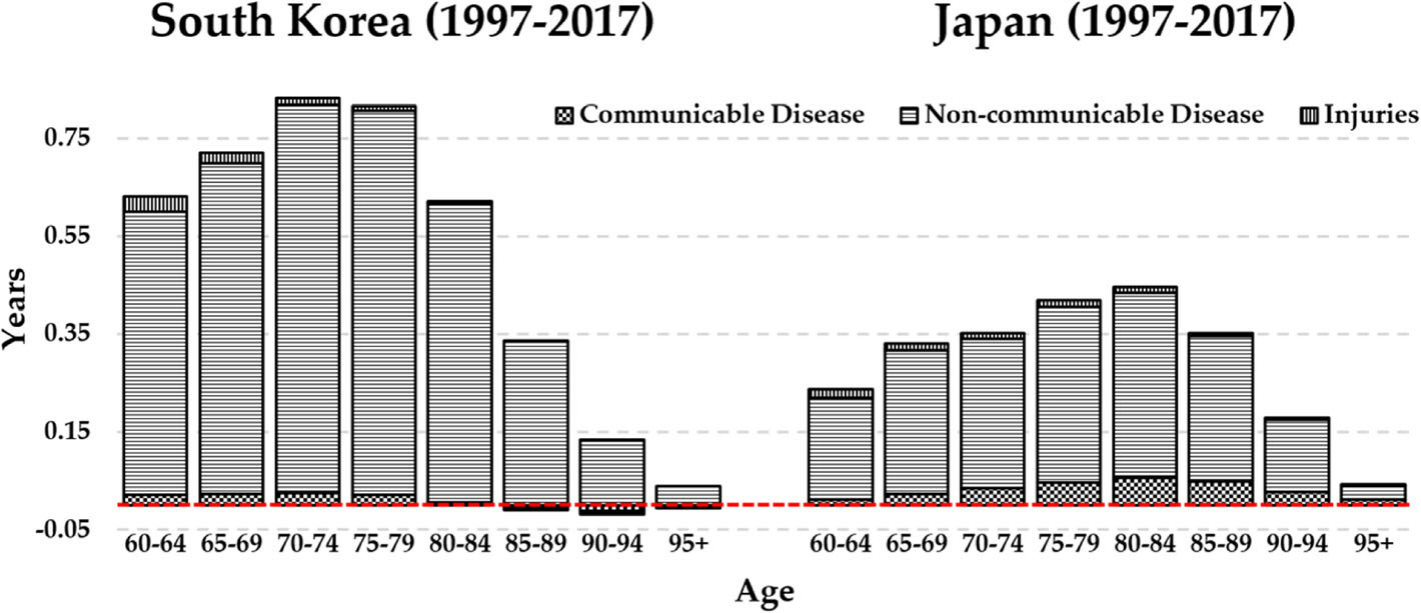
Age and cause specific contribution to the changes in old-age life expectancy of South Korea and Japan between 1997 and 2017

Life expectancy at 60 years increased by 5 years in South Korea and 2.53 years in Japan between 1997 and 2017. Table 3, columns 1 and 2 show the age-specific contributions to the changes in old age life expectancy in South Korea and Japan between 1997 and 2017. The contributions to the 5 year life expectancy gains in South Korea were from across all old age groups, but the largest contribution was observed in the 70–79 age group in South Korea. Life expectancy gains for Japanese old adults also occurred across all old age groups, but the largest improvement was observed in the 75–84 age group. Table 4, columns 1 and 2 show the cause-specific contribution to the change in life expectancy in South Korea and Japan between 1997 and 2017. Reduced mortality from stroke and ischaemic heart disease explained most of the increase in life expectancy and mortality reductions from cancers also explained a large part of the increase in both countries. In relation to the ASMRs, increased mortality from lower respiratory tract disease, self-harm and falls slightly led to decreasing life-expectancy of South Korean old adults between 1997 and 2017. In addition, the increased mortality from Parkinson’s disease and pancreatic cancer in South Korea and Japan, respectively, between 1997 and 2017 led to the marginal decreases in life expectancy of South Korean and Japanese old adults. Figure 3 depicts age- and cause-specific contributions of aggregate figures of 20 causes within the Level 1 category to the changes in life expectancy of Korean and Japanese old adults between 1997 and 2017. It shows that large improvements were found in the 70–79 age groups in South Korea while improvements were pronounced in the 75–84 age groups in Japan. The figure also clearly illustrates that, among 20 causes, reduced mortality from non-communicable disease greatly contributed to the increases in life expectancy at 60 years in both countries between 1997 and 2017; however, the contribution of communicable diseases to the increases was larger in Japan.

**Table 3:** Age specific contributions to the changes in life expectancy in South Korea and Japan

Age	Gap in Korea LE Between 1997 and 2017		Gap in Japan LE Between 1997 and 2017		Gap in LE between Korea and Japan in 1997 (a)		Gap in LE between Korea and Japan in 2017 (b)		Changes in gaps between 2017 and 1997 (b-a)	
	Years	%	Years	%	Years	%	Years	%	Years	%
60–64	0.74	14.8	0.27	10.7	0.47	12.4	−0.02	−1.5	− 0.49	19.8
65–69	0.83	16.6	0.37	14.6	0.52	13.7	0.04	3.0	−0.48	19.4
70–74	0.96	19.2	0.39	15.4	0.74	19.5	0.16	12.1	−0.58	23.5
75–79	0.96	19.2	0.45	17.8	0.79	20.8	0.28	21.2	−0.51	20.6
80–84	0.79	15.8	0.47	18.6	0.64	16.9	0.33	25.0	−0.31	12.6
85–89	0.47	9.4	0.36	14.2	0.40	10.6	0.31	23.5	−0.09	3.6
90–94	0.20	4.0	0.18	7.1	0.20	5.3	0.23	17.4	+ 0.03	−1.2
95+	0.05	1.0	0.04	1.6	0.03	0.8	−0.01	−0.70	−0.04	1.7
**Total**	**5.00**	**100**	**2.53**	**100**	**3.79**	**100**	**1.32**	**100**	**−2.47**	**100**

**Table 4:** Cause-specific contributions to the changes in life expectancy in South Korea and Japan

Cause of Death	Gap in Korea LE Between 1997 and 2017		Gap in Japan LE Between 1997 and 2017		Gap in LE between Korea and Japan in 1997 (a)		Gap in LE between Korea and Japan in 2017 (b)		Changes in gaps between 2017 and 1997 (b-a)	
	years	%	years	%	years	%	years	%	years	%
**Non-communicable disease**	**3.97**	**79.4**	**2.02**	**79.8**	**3.11**	**82.1**	**1.16**	**87.9**	**−1.95**	**78.9**
Stroke	1.72	34.4	0.66	26.1	1.36	35.9	0.26	19.7	−1.1	44.5
Stomach cancer	0.45	9.0	0.21	8.3	0.26	6.9	−0.02	−1.5	−0.28	11.3
Ischaemic heart disease	0.65	13.0	0.54	21.3	0.24	6.3	0.01	0.8	−0.23	9.3
Cirrhosis / liver diseases	0.27	5.4	0.03	1.2	0.26	6.9	0.03	2.3	−0.23	9.3
Tracheal / lung cancer	0.16	3.2	0.06	2.4	0.16	4.2	0.08	6.1	−0.08	3.2
Pancreatic cancer	0.01	0.2	−0.02	−0.8	− 0.01	−0.3	− 0.05	−3.8	−0.04	1.6
Diabetes mellitus	0.18	3.6	0.03	1.2	0.36	9.5	0.32	24.2	−0.04	1.6
Chronic obstructive pulmonary disease	0.16	3.2	0.08	3.0	0.18	4.7	0.14	10.6	−0.04	1.6
Liver cancer	0.16	3.2	0.15	5.9	0.12	3.2	0.11	8.3	−0.01	0.4
Hypertensive-heart disease	0.07	1.4	0.04	1.6	0.09	2.4	0.08	6.1	−0.01	0.4
Colon / rectal cancer	0.01	0.2	0.04	1.6	−0.07	−1.8	− 0.07	−5.3	−0.00	0.1
Gallbladder cancer	0.05	1.0	0.05	2.0	0.02	0.5	0.02	1.5	−0.00	0.1
Parkinson’s disease	−0.01	− 0.2	0.00	0.0	0.01	0.3	0.01	0.8	−0.00	0.0
Chronic kidney disease	0.03	0.6	0.09	3.6	−0.02	−0.5	0.00	0.0	+ 0.02	−0.8
Alzheimer / dementias	0.06	1.2	0.06	2.4	0.15	4.0	0.24	18.2	+ 0.09	−3.6
**Communicable disease**	**0.07**	**1.4**	**0.26**	**10.3**	**−0.13**	**−3.4**	**−0.04**	**− 3.0**	**+ 0.09**	**− 3.6**
Tuberculosis	0.16	3.2	0.04	1.6	0.17	4.5	0.07	5.3	−0.1	4.1
Lower respiratory tract	−0.09	−1.8	0.22	8.7	−0.3	−7.9	−0.11	−8.3	+ 0.19	−7.7
**Injuries**	**0.07**	**1.4**	**0.07**	**2.8**	**0.18**	**4.7**	**0.25**	**18.9**	**+ 0.07**	**−2.8**
Road injuries	0.15	3.0	0.04	1.6	0.16	4.2	0.1	7.6	−0.06	2.4
Falls	−0.01	−0.2	0.01	0.4	0.02	0.5	0.04	2.9	+ 0.02	−0.8
Self-harm	−0.07	−1.4	0.02	0.8	0.00	0.0	0.11	8.3	+ 0.11	−4.5
**Others**	**0.89**	**17.8**	**0.18**	**7.1**	**0.63**	**16.5**	**−0.05**	**−3.8**	**−0.68**	**27.5**
**Total**	**5.00**	**100**	**2.53**	**100**	**3.79**	**100**	**1.32**	**90.9**	**−2.47**	**100**

### Age- and cause-specific contributions to decreasing gaps in old age life expectancy between countries

**Fig. 4:**
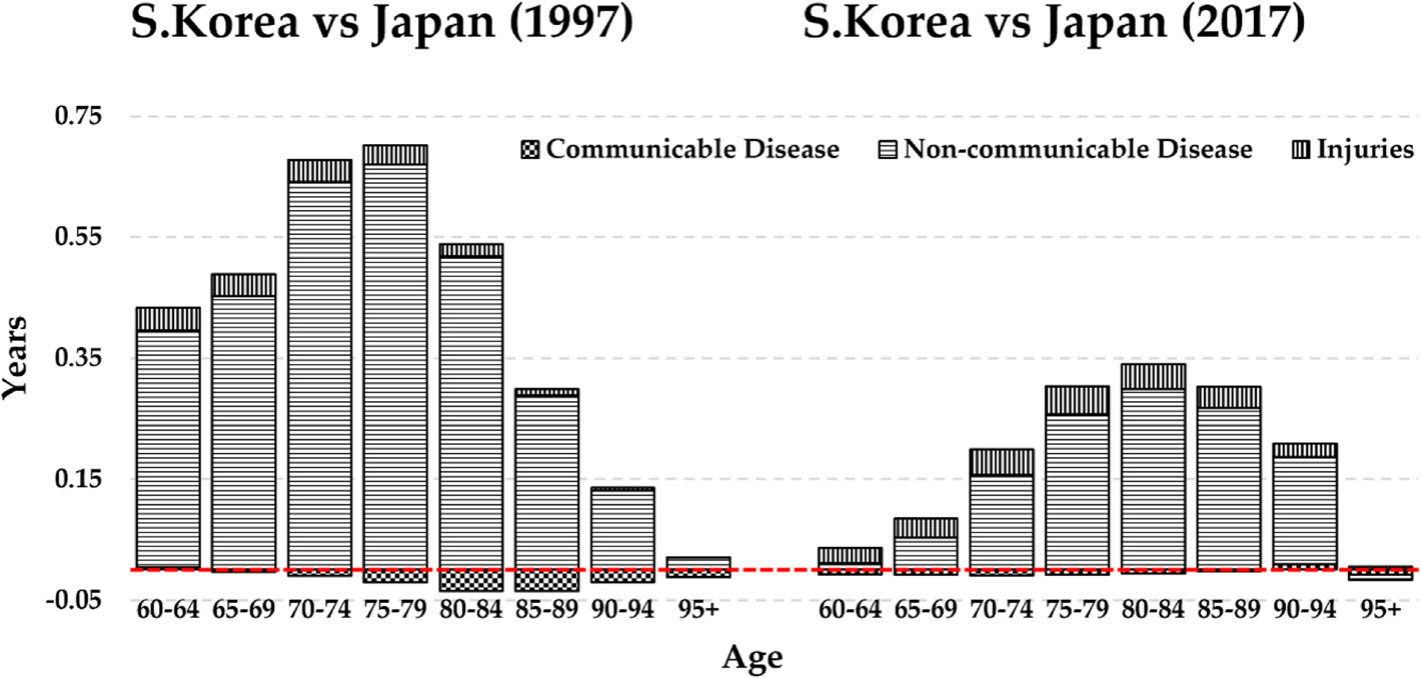
Age and cause specific contribution to the gaps in old-age life expectancy between South Korea and Japan in 1997 and 2017

Between 1997 and 2017, life expectancy gaps at 60 years of age between South Korea and Japan decreased by 2.47 from 3.79 in 1997, and 1.32 in 2017. Table 3, column 5 shows the age-specific contributions to the changes in gaps in life expectancy at 60 years between the two countries during 1997 and 2017, where negative figures indicate falls in gaps in old-age life expectancy. The age-specific contributions to the decreased gaps were found across all age groups, except for a small increase in the 90–94 age group, and the decreased gaps were especially large in the 70–79 years age group. Table 4, column 5 shows the cause-specific contributions to the decreased gap in life expectancy at 60 years in the two countries over the time period of 1997–2017, where positive signs indicate rises in gaps in old age life expectancy. Among 20 causes, cardiovascular disease (stroke, ischaemic heart disease and hypertensive-heart disease) and cancer deaths (stomach, liver, lung, pancreatic cancers), which were leading causes of the decline in ASMRs in South Korea between 1997 and 2017, contributed to the decreased gap in old-age life expectancy by 1.34 and 0.41, respectively. On the other hand, the three causes that showed increased ASMRs in South Korea between 1997 and 2017, lower respiratory tract disease, self-harm and falls, contributed to increased gaps in old age life expectancy between the two countries over the period. Moreover, changes in mortality pattern from two additional causes, chronic kidney disease and Alzheimer/Dementia, increased the gaps between the two countries over the period. However, the increased figure in chronic kidney disease (0.02 years) does not mean an increase in the gap, as the gap between these countries became almost zero in 2017, from a negative figure in 1997. In contrast, Alzheimer’s disease and dementia, which was the highest ASMRs for both countries in 2017, posed negative impacts on the gap between the two countries by 0.09 years during 1997–2017. Figure 4 depicts age- and cause-specific contributions of the aggregate figures of 20 causes within the IHME’s Level 1 category to the gaps in life expectancy at 60 years between the two countries during 1997 and 2017. Among the 20 causes, decreased gaps by non-communicable diseases were profound between 1997 and 2017 across all old age groups. However, bars indicating injury-associated causes slightly expanded from 1997 to 2017, especially in the age groups after 75. Moreover, although the gaps in communicable diseases decreased from − 0.13 to − 0.04, it should be noted that ASMRs of lower respiratory tract disease at an old age followed opposite trends in South Korea and Japan. In other words, ASMRs of lower respiratory tract disease among South Korean old adults is likely to outstrip Japan in the future. Overall, the result showed that causes from non-communicable diseases (cardiovascular disease and cancers) greatly contributed to decreasing the gaps in old-age life expectancy between South Korea and Japan; however, certain causes from communicable disease and injuries, in line with increased mortality patterns in South Korea, led to increasing the gaps.

## Discussion

This study explored the age- and cause-specific contributions to the changes in life expectancy at 60 years between Japan, the current life expectancy leader, and South Korea, the potential future leader, during 1997 and 2017. The study showed that age- and cause-specific contributions to the changes in old-age life expectancy differed in the two high-income Asia-pacific countries, and the result revealed that the decreasing gaps between the two countries were largely due to the mortality reductions in non-communicable diseases.

These findings are consistent with the epidemiological transition theory and other studies demonstrating that mortality in high-income countries is increasingly related to cardiovascular diseases (CVD) and cancers and, additionally, the average age of death from these diseases has shifted into older age with effective health prevention [4, 23]. This study showed that, in terms of age-specific contributions, the gaps in life expectancy among the elderly in the two countries were the largest in their 70’s in 1997 but in their 80’s in 2017, partly implying that South Korea experienced postponement of deaths at an older age [9, 23–25]. In terms of cause-specific contributions, both countries had the high ASMRs of CVD (stroke, ischaemic heart disease) and cancers in both 1997 and 2017, but these causes also explained considerable parts of both the increase in old-age life expectancy within the two countries, and the decrease in the gaps in old-age life expectancy between the two countries [9, 11, 26].

This study also showed that the considerable contributions of CVD and cancers to the decreased gaps in old-age life expectancy between the two countries were largely related to the reduced ASMRs of CVD and cancers in South Korea. This result coincides with Olshansky and Ault’s argument that death rates from degenerative diseases such as cancer and stroke rapidly decreased with effective and better healthcare services, as a country advances into, what they referred to, *‘the fourth stage of the epidemiologic transition: the age of delayed degenerative diseases*’ [27]. Previous research also showed that excellent health outcomes for old people in South Korea were primarily the result of effective health system performance through universal health coverage [28–31]. Particularly, targeting the four major causes of death, i.e., cancer, cerebrovascular, cardiovascular and rare and incurable diseases, was a salient public health intervention to reduce or delay mortality among old adults in South Korea, with priority given to these four major diseases through the National Health Insurance (NHI) benefit package [22] including the National Health Screening Programme (NHSP) and the National Screening Programme for Transitional Ages (NSPTA) [32, 33]. Therefore, the decreased gaps in old-age life expectancy between South Korea and Japan could be mainly the result of decreased or delayed mortality of Korean older adults from CVD and cancers by targeted public health interventions in South Korea.

Although the epidemiological transition theory provides the theoretical background for the decreased gaps in life expectancy among the elderly between the two countries, any explanation of increases in the gaps between the two countries due to self-harms and falls, lower respiratory tract disease, Alzheimer’s disease and dementia is incomplete. These causes may be a minor factor in the overall life expectancy; however, they are found in all three Level 1 classifications of cause of death (communicable, non-communicable disease and injuries). Furthermore, the increased gaps in old-life expectancy between South Korea and Japan resulted from three patterns of ASMRs from those causes. First, despite the similar levels of ASMRs of self-harm and falls in the two countries in 1997, in 2017 South Korea’s ASMRs increased, whereas Japan’s ASMRs decreased. Second, despite the overall higher ASMR of lower respiratory tract disease in Japan between 1997 and 2017, South Korea’s ASMR of lower respiratory tract disease increased, while Japan’s ASMR decreased. Third, ASMRs of Alzheimer’s disease and dementia increased in both countries between 1997 and 2017, but increased faster in South Korea. Although the main reasons behind the increase in the gaps from these patterns are undoubtedly multifactorial, rapid population ageing in South Korea may be the single most important factor.

With regard to elderly mortality increases from self-harm and falls in South Korea, this is partly attributed to combined effects of South Korea’s rapid family structural changes and population aging. For example, the proportion of the elderly population living alone in South Korea rose almost two-fold from 17% in 1990 to 33% in 2015 [34], whereas the corresponding figure only increased moderately from 11% in 1990 to 18% in 2015 in Japan [35]. Previous studies showed that the elderly living alone experienced more suicidal ideation and had a higher risk of falls than those who live with their spouse [36–38], suggesting that the faster increase in the number of older adults living alone in South Korea resulted in a higher risk of self-harms and falls. In addition to the slower increase in older adults living alone in Japan, ASMRs of self-harms and falls in Japan may have declined due to the beneficial impact of a range of community-based interventions such as depression screening, psychiatrist follow-up of old adults and fall prevention programmes [39–43].

With regard to lower respiratory tract disease, the results showed that ASMRs of lower respiratory tract disease were higher in Japan than South Korea in both years, but the two countries had opposite trends. It is well documented that lower respiratory tract disease and pneumonia in high-income countries are more associated with old people and, increasingly, with the Nursing Home and Healthcare Associated Pneumonia (NHCAP) due to population ageing, in contrast to a large prevalence of Community Associated Pneumonia (CAP) among the younger population in low-income countries [44, 45]. In Japan, the overall higher ASMR of lower respiratory tract disease could be largely attributed to NHCAP, since pneumonia is the third leading cause of mortality in Japan, with 97% of these deaths occurring in elderly patients over 65 years old [46]. Thus, the Japan Respiratory Society (JRS) in 2011 documented a new category of guidelines for NHCAP in order to manage the treatment of NHCAP, particularly among elderly residents in a long-term care hospital or a nursing home [47]. In South Korea, following the population ageing and the introduction of Long-Term Care Insurance, there has been a considerable increase in the number of Long-Term Care (LTC) facilities, from 1,754 in 2008 to 5,242 in 2017 [48]; however, this increase in LTC providers has been accompanied by service quality issues in South Korea [49]. Consequently, pneumonia is now the leading cause of death among residents of long-term care facilities, and unfavorable institutional factors in long-term care facilities have often been reported as increasing prognostic factors for pneumonia [50–52].

With regard to Alzheimer’s disease and dementia, the observed increase in the pattern between 1997 and 2017 in both countries should be interpreted carefully, in particular due to the difficulties in reporting dementia and Alzheimer’s disease [53]. First, there might have been underreporting of dementia and Alzheimer’s disease as the cause of death in 1997 due to a lack of diagnostic tools and awareness of people in South Korea and Japan in 1997. Second, the larger number of deaths caused by Alzheimer’s disease and dementia in 2017 might have been partially due to more accurate death reporting and registration because of improved diagnostic tools and aweareness. This study does not attempt to discuss reporting accuracy; nonetheless, it is plausible to think that the combination of faster population ageing and increased awareness of the disease in South Korea may have led to more cases of Alzheimer’s disease and dementia in South Korea. Indeed, both countries proposed a series of plans to promote the community-based integrated care system for the elderly with dementia such as the New Orange Plan (2015) in Japan and the third National Dementia Management Master Plan 2016–2020 (2015) in South Korea. However, much faster population ageing and growing awareness of Alzheimer’s disease and dementia in South Korea may increase public health and social burdens in the country for the next coming years.

The first limitation of this study is that it only focused on the top 20 causes of death based on South Korea in 2017, and thus the emerging causes of death among the elderly in both countries may have been missed. Secondly, although life expectancy is a valid indicator of a population’s health status, this study cannot tell whether old adults in both countries lived longer and healthier lives or simply experienced extended periods of morbidity. Further studies to explore healthy life expectancy are required. Lastly, this case study investigated only two ageing countries in the Asia-pacific region. More comparative studies of increasing or decreasing gaps in life expectancy between other high or middle-income Asia-pacific countries confronting a possible double burden of the increasing threat of non-communicable disease in parallel with emerging communicable disease due to demographic ageing should be carried out. Many countries in the Asian Pacific region are also experiencing accelerated population ageing, and therefore their governments are trying to prepare for sustainable health-care systems in response to the inevitable ageing of the population. This comparative study between high- income Asia-pacific countries in terms of cause-specific mortality and life expectancy can provide insights into how to relieve the future health burden associated with population ageing in countries in the Asian Pacific region.

Taken together, old-age life expectancy can reflect health and wellbeing of an elderly population and investigating gaps in old-age life expectancy between countries can facilitate cross-national policy learning in an era of demographic ageing. This comparative study showed that age- and cause-specific contributions to the changes in old-age life expectancy can differ between two high-income and ageing countries. Moreover, although mortality changes in non-communicable diseases was a key influencing factor of decreasing gaps in old-age life expectancy between the two countries, the decreasing gaps might also be disturbed by emerging threats of communicable disease and injuries along with rapid demographic ageing.

## Conclusions

This study affirms that age- and cause-specific contributions to the changes in old-age life expectancy can differ between high-income Asia-pacific countries. Although the gaps in old-age life expectancy between high-income Asia-pacific countries are primarily attributed to mortality changes in non-communicable diseases, these countries should also identify potential emerging threats of communicable diseases and injuries along with demographic ageing in pursuit of healthy life years in old age.

## Data Availability

All data used in this study is publicly available through websites the World Population Prospects (https://population.un.org/wpp/) and the Global Health Data Exchange (http://ghdx.healthdata.org).

## Abbreviations

ADL: Activities of Daily Living
ASMR: Age-standardised Mortality Rate
CAP: Community Associated Pneumonia
CMF: Comparative Mortality Figure
CMNN: Communicable, maternal, neonatal and nutritional disease
CVD: Cardiovascular disease
GBD: Global Burden Disease
IHME: The Institute for Health Metrics and Evaluation
LE: Life Expectancy
LTC: Long-Term Care
NCDs: Non-communicable diseases
NHCAP: Nursing Home and Healthcare Associated Pneumonia
NHSP: National Health Screening Programme
NSPTA: National Screening Programme for Transitional Ages
WPP: World Population Prospects
